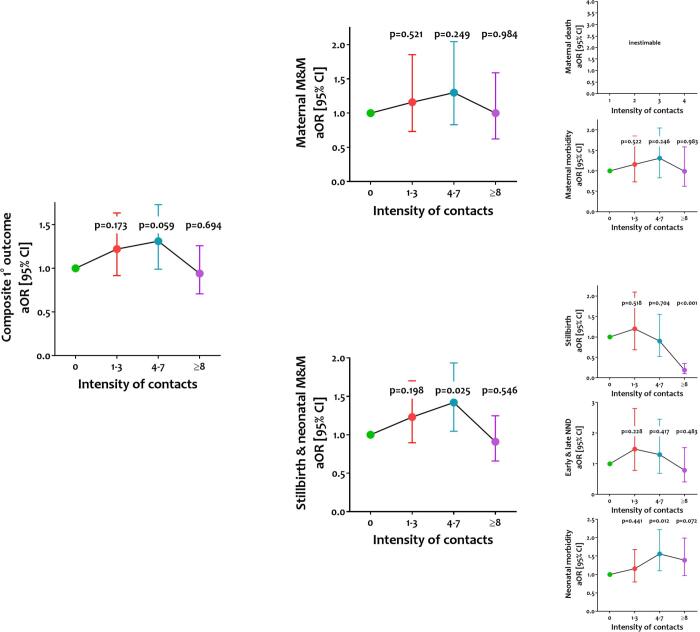# Community level interventions for pre-eclampsia (CLIP) in India: A cluster randomised controlled trial

**DOI:** 10.1016/j.preghy.2020.05.008

**Published:** 2020-07

**Authors:** Mrutunjaya B. Bellad, Shivaprasad S. Goudar, Ashalata A. Mallapur, Sumedha Sharma, Jeffrey Bone, Umesh S. Charantimath, Geetanjali M. Katageri, Umesh Y Ramadurg, J. Mark Ansermino, Richard J. Derman, Dustin T. Dunsmuir, Narayan V. Honnungar, Chandrashekhar Karadiguddi, Avinash J. Kavi, Bhalachandra S. Kodkany, Tang Lee, Jing Li, Hannah L. Nathan, Beth A. Payne, Amit P. Revankar, Andrew H. Shennan, Joel Singer, Domena K. Tu, Marianne Vidler, Hubert Wong, Zulfiqar A. Bhutta, Laura A. Magee, Peter von Dadelszen, the CLIP India Working Group (Table S1)

**Affiliations:** a KLE Academy of Higher Education and Research’s J N Medical College, Nehru Nagar, Belagavi, 590010 Karnataka, India; b S Nijalingappa Medical College, HSK (Hanagal Shree Kumareshwar) Hospital and Research Centre, Navanagar, Bagalkot, 587102 Karnataka, India; c Department of Obstetrics and Gynaecology, Faculty of Medicine, University of British Columbia, Suite 930, 1125 Howe Street, Vancouver, BC V6Z 2K8, Canada; d Centre for International Child Health, 305 - 4088 Cambie Street, Vancouver V5Z 2X8, Canada; e Global Affairs, 1020 Walnut Street, Thomas Jefferson University, Philadelphia 19107, USA; f Department of Women and Children’s Health, School of Life Course Sciences, Faculty of Life Sciences and Medicine, King’s College London, St. Thomas’ Hospital, Westminster Bridge Road, London SE1 7EH, UK; g Centre for Health Evaluation and Outcome Sciences, Providence Health Care Research Institute, University of British Columbia, 588 - 1081 Burrard Street, St. Paul’s Hospital, Vancouver V6Z 1Y6, Canada; h Centre for Global Child Health, Hospital for Sick Children, 525 University Avenue, Suite 702, Toronto M5G 2L3, Canada; i Aga Khan University, Stadium Road, P.O. Box 3500, Karachi 74800, Pakistan

**Keywords:** Cluster randomized controlled trial, Pregnancy hypertension, India, Community engagement, Mobile health, Community health worker

## Abstract

**Objectives:**

Pregnancy hypertension is associated with 7.1% of maternal deaths in India. The objective of this trial was to assess whether task-sharing care might reduce adverse pregnancy outcomes related to delays in triage, transport, and treatment.

**Study design:**

The Indian Community-Level Interventions for Pre-eclampsia (CLIP) open-label cluster randomised controlled trial (NCT01911494) recruited pregnant women in 12 clusters (initial four-cluster internal pilot) in Belagavi and Bagalkote, Karnataka. The CLIP intervention (6 clusters) consisted of community engagement, community health workers (CHW) provided mobile health (mHeath)-guided clinical assessment, initial treatment, and referral to facility either urgently (<4 h) or non-urgently (<24 h), dependent on algorithm-defined risk. Treatment effect was estimated by multi-level logistic regression modelling, adjusted for prognostically-significant baseline variables. Predefined secondary analyses included safety and evaluation of the intensity of mHealth-guided CHW-provided contacts.

**Main outcome measures:**

20% reduction in composite of maternal, fetal, and newborn mortality and major morbidity.

**Results:**

All 14,783 recruited pregnancies (7839 intervention, 6944 control) were followed-up. The primary outcome did not differ between intervention and control arms (adjusted odds ratio (aOR) 0.92 [95% confidence interval 0.74, 1.15]; p = 0.47; intraclass correlation coefficient 0.013). There were no intervention-related safety concerns following administration of either methyldopa or MgSO_4_, and 401 facility referrals. Compared with intervention arm women without CLIP contacts, those with ≥8 contacts suffered fewer stillbirths (aOR 0.19 [0.10, 0.35]; p < 0.001), at the probable expense of survivable neonatal morbidity (aOR 1.39 [0.97, 1.99]; p = 0.072).

**Conclusions:**

As implemented, solely community-level interventions focussed on pre-eclampsia did not improve outcomes in northwest Karnataka.

## Highlights

• As implemented, the CLIP intervention did not improve the primary composite outcome.
• ASHAs and ANMs were able to undertake all aspects of the mobile health app-guided visits.
• Women could not be reached in their communities as frequently as planned.
• Eight or more POM-guided contacts were associated with fewer stillbirths supporting WHO guidance.
• Community-level interventions are unlikely to improve outcomes without enhanced facility care.

## 1 Introduction

India bears the greatest burden of maternal, fetal, and infant deaths [1]. Within India, there remain disparities in access to health care and death rates across states and between urban and rural populations [2].

Hypertension, complicating 10.3% of pregnancies in northwest Karnataka [3], is associated with excess maternal, fetal and newborn deaths and morbidities [4], potentially amenable to reductions through standardised care [5], [6].

Communities report regular use of health care services during pregnancy (generally four antenatal care (ANC) visits) and for delivery [7]. However, delays in care-seeking, such as declaring pregnancy or attending first antenatal visits, persist; community-based initiatives to encourage early disclosure of pregnancies and facility-based care could address these delays. Also, there are significant knowledge gaps in community perceptions of the causes, warning signs, and consequences of pregnancy hypertension [8]. There are local words in Kannada for ‘convulsions’ and ‘hypertension’, but there are no pregnancy-specific terms. Stress, tension, and poor diet are perceived to precipitate pregnancy hypertension, while anaemia, poor medical adherence, lack of tetanus toxoid immunisation, and exposure to fire or water are perceived precipitants of seizures in pregnancy [8]. Sweating, fatigue, dizziness-unsteadiness, swelling, and irritability are perceived as signs of hypertension [8]. While hypertension is a recognised risk factor for both eclampsia and maternal death, home remedies are used regularly to treat seizures prior to accessing facility-based care [7], [8].

Therefore, there is potential to improve outcomes by both community engagement and community-based assessment, triage, and initial treatment by India’s two main cadres of community health workers (CHWs), Accredited Social Health Activists (ASHAs) and Auxiliary Nurse Midwives (ANMs). ASHAs, local women who receive 23 days’ training, provide health education and promotion to ≈1000 people in their communities [9]. In contrast, ANMs and staff nurses are responsible for providing maternal and child health services, primarily in sub-centres and primary health centres (PHCs), each serving a catchment of 3000–5000 people.

The objective of the Community-Level Interventions for Pre-eclampsia (CLIP) India cluster randomised controlled trial (cRCT) was to test the hypothesis that implementing community-level, evidence-based care focussed on pregnancy hypertension would reduce all-cause maternal, fetal and newborn mortality and major morbidity, without causing harm [10].

## 2 Methods

The full protocol has been published (https://clinicaltrials.gov/ct2/show/NCT01911494 and Appendix 1), and received ethics approvals (KLE University (MDC/IECHSR/2011-12/A-4; ICMR 5/7/859/12-RHN); University of British Columbia (UBC, H12-03497)).

### 2.1 Study setting and trial design

**Fig. 1:**
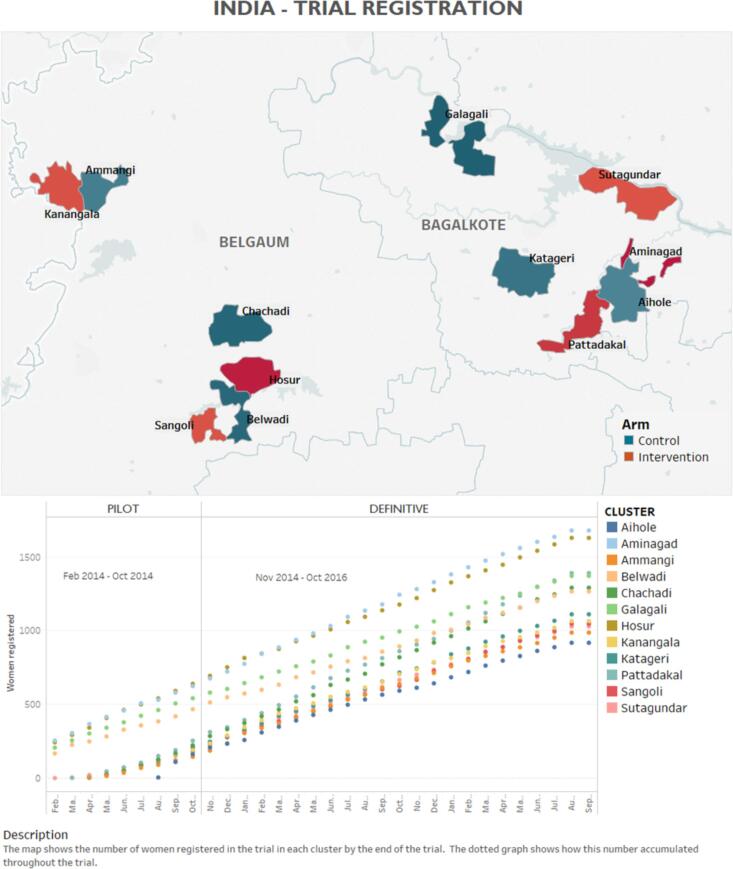
Map of study area and enrollment by cluster.

The CLIP India Trial, one of three independently-powered open-label cRCTs (others in Pakistan and Mozambique; all NCT01911494), occurred in Belagavi (1278 villages) and Bagalkote (627 villages) districts, rural Karnataka (Fig. 1), with a population density of ≈13 persons/ha. Adult female literacy rates vary (Belagavi: 65%; Bagalkote: 58%) [11]. Operationally-active key policy factors included *Janani Suraksha Yojna* (conditional cash-transfer programme promoting institutional deliveries), and provision of *Madilu* kits following government facility deliveries [12].

A 12-month mixed methods feasibility study preceded the internal pilot phase (1 February to 31 October 2014) in four clusters [7], [8], [9], [13], [14], [15], [16]. Progression to the definitive phase (additional eight clusters (1 November 2014 to 31 October 2016)) was based on the criterion of ≥50% acceptance of CLIP-indicated health facility referral.

### 2.2 Randomisation

The unit of randomisation was the cluster, each defined as a PHC, and its catchment area; each being chosen according to region (Belagavi or Bagalkote), accessibility for surveillance, and absence of conflicting research activity. The 12 clusters were allocated (TL, statistician) to intervention or control groups using a restricted randomisation algorithm, balancing region (Belagavi or Bagalkote) and population size. For the four internal pilot phase clusters, one of two possible allocations meeting the balancing criteria was randomly selected. Similarly, for the definitive phase, one of 14 possible allocations for the additional eight clusters (pilot cluster allocations were fixed) was randomly selected.

### 2.3 Participants

Participants were married pregnant women, aged 15–49 years, who were either resident (≥1 year) or non-resident, but planning to deliver in the cluster. Enrolled women who migrated out of the cluster during pregnancy were followed-up. All women (intervention and control) provided written, informed consent for data collection in a health registry. Intervention cluster women provided additional intervention-specific written consent. The trial surveillance team informed cluster ANMs and ASHAs about eligible and consented pregnant women with registration data. Recruitment ended on 31 August 2016 to permit follow-up to delivery.

### 2.4 Interventions

**Supplementary figure 1:**
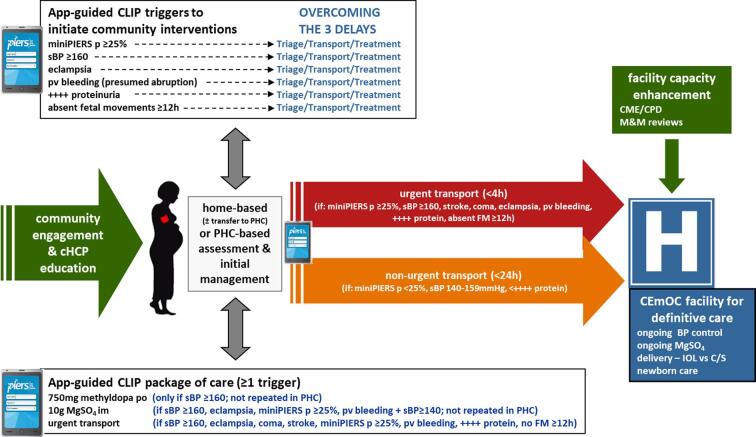


In intervention clusters, the CLIP intervention package consisted of both community engagement and CHW-led contacts in eligible women’s homes and PHCs, focussing on detecting and managing pregnancy hypertension (Fig. S1 and Table S2).

Community engagement involved: community leaders; pregnant women, their mothers, mothers-in-law, husbands and other stakeholders. The content promoted: (i) awareness of signs, symptoms, and potential consequences of pre-eclampsia and eclampsia; (ii) education about birth preparedness and complication readiness, including prior permissions for care-seeking, transport plans and funds for obstetric emergencies; and (iii) the nature of CLIP visits using the PIERS On the Move (POM) tool [17], [18], [19] and the CLIP package of care, as relevant (see below). PHC medical officers, staff nurses, and ANMs were trained to conduct community engagement sessions with defined topics and supported by pictograms developed with local input, which occurred from the time of cluster randomisation until the end of trial.

CHW-led ‘CLIP’ antenatal contacts were recommended to occur: every four-weeks (<28 weeks), fortnightly (28–35 weeks), weekly (36 weeks until birth), <24 h of birth and on postpartum days 3, 7, and 14; the compliance target was one CLIP contact every six weeks. Home-based contacts were provided by ASHAs, or, where not available, Anganwadi Workers (community workers). PHC-based visits were provided by ANMs and staff nurses.

CLIP contact tasks were guided by the mobile-based CLIP POM mobile health (mHealth) application (app) [18], that included the miniPIERS (Pre-eclampsia Integrated Estimate of RiSk) time-of-disease tool [19], with pictograms as visual prompts. The app directed workers to first observe women to exclude emergency conditions warranting immediate facility referral. In the absence of emergency conditions, CHWs were directed to measure blood pressure (BP) twice, using standardised methods and a semi-automated, pregnancy-validated digital device (Microlife BP 3AS1-2) [20]; a third measurement was required if there was a between-reading difference in either systolic or diastolic BP > 10 mmHg. Special government permissions for CHWs to measure BP and test for proteinuria were granted. All de-identified data were synchronised directly from the POM mobiles to the central REDCap server for data monitoring. Each visit was time stamp validated.

148 health workers were trained to provide the CLIP intervention: 102 ASHAs, seven Anganwadi Workers, 21 ANMs, and 18 staff nurses. Two-day didactic and participatory training consisted of BP measurement, proteinuria assessment, and POM app use (six-monthly refresher training). ASHAs and Anganwadi workers were trained to bring women recommended by POM to receive community-based treatment to PHCs, where trained ANMs and staff provided POM-indicated oral antihypertensive therapy and intramuscular MgSO_4_ (Fig. S1 and Table S2).

For normotensive well women, the app directed ASHAs and ANMs to continue routine CLIP contacts at the recommended frequency (see above).

For hypertensive women, app-directed ASHAs and ANMs entered observations about pre-eclampsia symptoms and dipstick proteinuria into the POM app. Embedded decision algorithms risk-stratified women and directed CHWs to recommend appropriate care: initial management with 750 mg oral methyldopa (severe hypertension) or 10 g intramuscular MgSO_4_ (pre-eclampsia with adverse features (e.g., miniPIERS score > 25% or severe systolic hypertension ≥ 160 mmHg)), and referral to facility either urgently (<4 h) for an emergency condition, severe pre-eclampsia, or decreased fetal movement, or non-urgently (<24 h) for systolic BP 140–159 mmHg without evidence of severe pre-eclampsia or decreased fetal movement (Fig. S1 and Table S2).

In control clusters, women received routine ANC provided at PHCs by nurses and doctors.

In both intervention and control clusters, health care providers at secondary and higher facilities (private and public) received training (three sessions) to promote evidence-based care of women with pre-eclampsia/eclampsia.

### 2.5 Procedures for surveillance

A prospective population-based surveillance system was established [15], modelled on the National Institute of Health Global Network’s Maternal and Newborn Health (MNH) Registry [15]. In brief, data were collected in the local language by cluster PHC health care workers on three standard MNH Registry forms, three supplemental forms covering additional CLIP-specific fields, and modified WHO verbal autopsy forms for maternal, fetal, or newborn deaths. Data were obtained by reviewing facility medical records (when available) and interviews (women, birth attendants, health care providers, and family members) [21]. Completed paper data forms were keyed weekly into the database. Completeness was confirmed by separate trained personnel. De-identified and encrypted data were transferred to the UBC central server for analyses.

Married women of reproductive age predicted to conceive within 12 months were identified by annual household survey, followed up for early pregnancy confirmation, and consented and enrolled into the CLIP trial once pregnant. Data were collected at three time points: (i) soon after enrolment; (ii) soon after delivery; and (iii) 42 days postpartum.

### 2.6 Outcomes

The primary outcome was a composite of all-cause maternal, fetal, and neonatal mortality and major morbidity. Pregnancies with multiple elements of the primary outcome were only counted once without weighting. Mortality was assessed until 42 (mother) and 28 (offspring) days after birth and described per 1000 identified pregnancies. Maternal morbidity consisted of serious end-organ complications that included, but were not limited to, those related to hypertensive pregnancy, as the CLIP intervention could have had a generally beneficial effect. Neonatal morbidity reflected problems related to early delivery or delivery of a baby in poor health. All deaths, as well as survived morbidities, were reviewed by a committee masked to cluster of origin and excluding practitioners who cared for the individual under review.

The secondary outcomes were birth preparedness and complication readiness [7], [22]; delivery in facility able to provide emergency obstetric care; proportion of facility births; and intervention-related adverse events. Other outcomes included the impact of the intensity of contacts on the incidence of the primary outcome and its components.

For outcome definitions, see Panel 1.

### 2.7 Sample size

We estimated a requirement for 12 clusters based on the following assumptions: (i) baseline incidence of our composite primary outcome of 5.4% (based on a maternal mortality ratio of 150/100,000 livebirths [21], and assuming a 5:1 ratio of maternal near-miss events and a subsequent 5:1 ratio of perinatal to maternal events); (ii) a 20% reduction in our primary outcome in intervention vs. control clusters; (iii) 10% loss to follow-up; (iv) an annual birth rate of 22/1000/year/cluster; and (v) an intra-cluster co-efficient (ICC) of 0·001 based on previous local experience [23]. The sample size was calculated by simulations of 5000 Monte Carlo samples based on the input parameters. Power was calculated as the proportion of times within the 5000 samples that the difference in the primary composite outcome between intervention and control cluster reached statistical significance (p < 0.05).

### 2.8 Statistical analysis (full plan, Appendix 2)

All pregnancies, except withdrawals, were included in our primary, intention-to-treat analyses. The unit of analysis was pregnancy, classified as ‘followed-up’ (complete postpartum trial surveillance), ‘lost-to-follow-up’ (estimated date of delivery [EDD] at ≥3 weeks before trial end), and ‘still on follow-up’ (EDD < 3 weeks before trial end).

To mitigate potential bias due to differential loss-to and incomplete follow-up, the primary outcome (and its components) of women who were lost-to, or still-on, follow-up was imputed 10 times (multiple imputation by chain equations) and pooled (Rubin’s rules) [24]. Imputation models were based on all primary analysis adjustment factors (see below) and interactions between trial arm and enrolment date. In each imputed data set, the adjusted odds ratio (OR) for the intervention effect was estimated using a multi-level logistic regression including a random intercept term for each cluster. To improve precision, models were adjusted for variables at individual (i.e., age, parity, maternal primary education, previous delivery locations, and husband’s primary education) and cluster-level (i.e., baseline neonatal mortality rate and population density). When it was observed that the ICC was higher than anticipated in the internal pilot phase, baseline neonatal mortality rate was added as an adjustment factor to improve power, which would not have been addressed by extending recruitment within clusters.

Analogous multi-level logistic regression models were fit to assess the sensitivity of the primary result to various factors, including adjustment, imputation, missing data for a component of the primary outcome (when others were documented), gestational age at birth, and postpartum follow-up to <42 days (Appendix 2), as well as cluster-level aggregate analysis. Where sensitivity analyses included imputation, results were pooled (Rubin’s rules). In an additional planned secondary analysis, we explored within the intervention arm, whether there was a relationship between our primary outcome and the intensity of CLIP visits, categorised as 0, 1–3, 4–7, or ≥8, to reflect prior and current WHO recommendations for the frequency of antenatal contacts [25]. To account for factors related to the number of POM-guided contacts and confounders, the analysis was adjusted for maternal age, basic education, parity, enrolment timing in the trial, and distance from the household to facility.

All analyses were repeated for each primary outcome component, albeit without imputation. Secondary and other outcomes were compared by baseline factor-adjusted multi-level models, as above.

Statistical significance (two-sided) was set at p < 0.05 for the primary and p < 0.01 for other analyses using ‘R’ software throughout, without adjustment for the interim analysis.

An interim analysis was undertaken once complete data were received for women making up half of the planned sample size and reviewed by the data safety and monitoring board (DSMB). The stopping rule for both benefit and harm required an observed difference between groups associated with an alpha <0.001 (power 80%). The DSMB reviewed all reported adverse events for participant safety.

## 3 Results

**Fig. 2:**
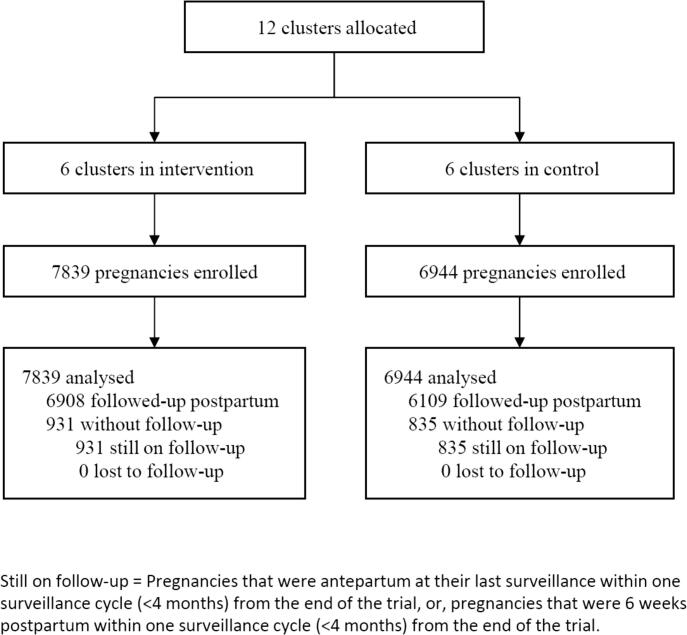
Trial profile – Intervention vs. control allocation clusters.

Between 1 February 2014 and 31 October 2016, 14,783 pregnancies were recruited in six intervention (7839 pregnancies) and six control (6944 pregnancies) clusters (Fig. 2); 1390 of these pregnancies were enrolled during the pilot phase (777 in two intervention and 613 in two control clusters). The criterion for progression from the internal pilot to definitive trial phase was met, according to 74% acceptance of referral to facility (49/66 pregnancies). One cluster was replaced (by another with similar characteristics) after allocation but before any recruitment or participation in the trial, in response to issues with PHC leadership and data integrity. There were no withdrawals and all pregnancies were included in the primary analysis.

Pregnancies in intervention and control clusters were similar at baseline (Table 1). The women were generally in their early 20 s and of Hindu religion, and just over half of them and their husbands had basic education. Women were generally parous and when so, not infrequently reported prior stillbirth (≈5%) or neonatal death (≈5%) despite almost universal facility deliveries. Few women (<1%) had multiple pregnancies. Women were usually enrolled in the first trimester and were found to be anaemic. Migration rates were similar (15.3% [intervention] vs. 17.5% [control]).

**Table 1:** Baseline characteristics.

	Intervention (n = 7839 pregnancies)	Control (n = 6944 pregnancies)
**Clusters**	6	6
Population density (n/ha)^a^	11.6	13.3
Estimated annual birth rate/cluster (per year)	22/1000	22/1000
ANMs/cluster (n)	4 [3.5–4.5]	3.5 [3–4]
ASHAs/cluster (n)	17 [16–18]	18 [17–21]
Neonatal mortality ratio/1000 live births (in last 12 months at baseline)^a^	20.5 (20.0–25.5)	20.5 (20.0–21.0)
**Enrolled pregnancies**	7839	6944
Maternal age (year)^a^	23.0 (20.0–25.0)	22.0 (20.0–25.0)
Religion^b^		
Hindu	7224 (92.2%)	6304 (90.8%)
Muslim	538 (6.9%)	637 (9.2%)
Other	77 (1.0%)	3 (0%)
Women with ≥8 years of schooling^a^	4541 (57.9%)	3872 (55.8%)
Husbands with ≥8 years of schooling^b^	4827 (61.6%)	4006 (57.7%)
Anaemia^b^	6455 (82.3%)	5955 (85.8%)
**Obstetric history**		
Parousa	4953 (63.2%)	4481 (64.5%)
Parity	1.0 (0.0–2.0)	1.0 (0.0–2.0)
Amongst previously pregnant women		
Had previous stillbirth(s)^b^^,^^c^	235 (4.5%)	263 (5.5%)
Had previous neonatal death(s)	284 (5.4%)	272 (5.7%)
Delivery location in previous pregnancy		
Home	533 (10.2%)	517 (10.9%)
CEmOC (hospitals)	2895 (55.4%)	2946 (62.1%)
Non-CEmOC facility	1797 (34.4%)	1270 (26.8%)
ANC care sought in previous pregnancy	6891 (99.8%)	6109 (99.3%)
**Current pregnancy**		
Gestational age at enrolment (week)	10.3 (7.7–14.1)	10.9 (8.0–15.1)
Multiple pregnancy	61 (0.9%)	41 (0.7%)

Data presented as median (interquartile range) or number (%).

ANMs = Auxiliary Nurse Midwives. ASHAs = Accredited Social Health Activists. CEmOC = Comprehensive emergency obstetric care.

a Variables used as adjustment factors in analyses, chosen *a priori*.

b Variable used as adjustment factor in analyses, chosen following review of baseline data prior to knowledge of outcomes.

c Not asked in pilot phase.

In the intervention arm, 1379 community engagement sessions (median 214.5 [IQR 192–266]/cluster) involving 39,619 participants (median 6742.5 [IQR 5736–7154]/cluster) were held. All sessions involved married women of reproductive age, 1140 (82.7%) involved mothers-in-law, and 1147 (83.2%) involved key stakeholders, primarily members of village councils (*gram panchayat*), and local health committees, partners, fathers, fathers-in-law, religious leaders, traditional health care providers and formal care providers (Table S3).

ASHA, ANM (and Anganwadi worker) coverage per cluster was consistent between clusters (range: 10.7 [Pattadkal (Bagalkote)] to 13.5 [Sangolli (Belagavi)]) (Table S3).

≥1 POM contact by a CHW (total: 63,992 contacts) was received in 7054 pregnancies (90.0%), for a median of 8 [IQR 4, 14] contacts when POM was received, and 84.9% were antenatal. The contact number compliance target was met in 2948 pregnancies (41.8%). Compliance with BP measurement at all contacts, and proteinuria testing during first and hypertensive visits, was almost uniform (Table S3). POM-guided recommendations for referral were accepted by most women; non-urgent referral (5.4%) was accepted 76.7% of the time. Urgent referral (1.8%), most commonly for severe hypertension (46.1%) or absent fetal movements in the preceding 24 h (22.7%), was accepted 84.9% of the time. Community-level initiation of treatment was recommended during <1.0% of visits, with acceptance of oral methyldopa for severe hypertension higher (84.9%) than intramuscular MgSO_4_ for pre-eclampsia (67.6%) (Table S3).

There was no difference in routine ANC for women in intervention (vs. control) clusters (aOR 2.87 99% CI =  (0.22, 37.69); p = 0.291).

The composite primary outcome (one or more element) complicated 162.8 per 1000 identified pregnancies, primarily due to major neonatal morbidity (Table 2). Major maternal morbidity (≈5%) was 43-fold more common than maternal death (1.1/1000 identified pregnancies). Stillbirths and neonatal deaths (both ≈20/1000 identified pregnancies) were less common than major neonatal morbidity (≈11%). The most common major maternal morbidity was blood transfusion (3.2%), followed by obstetric sepsis (0.9%), and antepartum haemorrhage (0.7%); others occurred in <0.5% of women. The most common major neonatal morbidity was breathing difficulty (6.6%), followed by lethargy (5.1%) and feeding difficulty (5.0%).

**Table 2:** Primary outcome.

	Intervention (n = 7839 pregnancies)	Control (n = 6944 pregnancies)	Adjusted OR^a^ (95% CI)	p-value
Pregnancies with postpartum follow-up	6908 (88.1%)	6109 (88.0%)	–	–
Infants	6968	6148	–	–
**Composite primary outcome**^b^	1252 (16.0%)	1154 (16.6%)	0.92 (0.74, 1.15)	0.47
Maternal mortality	7 (0.1%)	9 (0.1%)	0.59 (0.14, 2.77)	0.47
Maternal morbidity (including maternal deaths)	371 (4.7%)	325 (4.7%)	1.04 (0.76, 1.43)	0.80
Antepartum haemorrhage	68 (0.9%)	42 (0.6%)	–	–
Stroke	9 (0.1%)	4 (0.1%)	–	–
Obstetric sepsis	60 (0.8%)	74 (1.1%)	–	–
Maternal coma	3 (0.0%)	1 (0.0%)	–	–
Interventions for major postpartum haemorrhage	4 (0.05%)	6 (0.08%)		
Seizure	26 (0.3%)	21 (0.3%)	–	–
Fistula	0 (0.0%)	0 (0.0%)	–	–
Disseminated intravascular coagulation	4 (0.1%)	5 (0.1%)	–	–
Cardiopulmonary resuscitation	3 (0.0%)	1 (0.00%)	–	–
Blood transfusion	263 (3.4%)	215 (3.1%)	–	–
Mechanical ventilation	9 (0.1%)	2 (0.0%)	–	–
Dialysis	1 (0.0%)	1 (0.0%)	–	–
Perinatal mortality and late neonatal mortality	367 (4.7%)	292 (4.2%)	1.05 (0.89, 1.24)	0.56
Stillbirth	191 (2.4%)	156 (2.2%)	–	–
Early neonatal death	146 (1.9%)	106 (1.5%)	–	–
Late neonatal death	34 (0.4%)	31 (0.4%)	–	–
Neonatal morbidity	813 (10.4%)	790 (11.4%)	0.89 (0.67, 1.17)	0.39
Breathing difficulty	494 (6.3%)	486 (7.0%)	–	–
Lethargy	398 (5.1%)	353 (5.1%)	–	–
Feeding difficulty	379 (4.8%)	357 (5.1%)	–	–
Jaundice	154 (2.0%)	148 (2.1%)	–	–
Seizure	63 (0.8%)	62 (0.9%)	–	–
Umbilical cord infection	66 (0.8%)	37 (0.5%)	–	–
Coma	37 (0.5%)	23 (0.3%)	–	–
Hypothermia	11 (0.1%)	39 (0.6%)	–	–
Skin infection	23 (0.3%)	25 (0.4%)	–	–
Bleeding	8 (0.1%)	3 (0.0%)	–	–

Data presented as number (%) or number only.

CI = confidence interval. OR = odds ratio.

a Adjusted for individual-level factors (maternal age, parity, maternal education, anaemia, stillbirth in previous pregnancy, husband’s education, delivery location in previous pregnancy), and cluster-level factors (population density, baseline study neonatal mortality rate).

b Defined as one/more of maternal morbidity or mortality, stillbirth, neonatal mortality, or neonatal morbidity.

The primary outcome did not differ between intervention and control clusters either overall (aOR 0.92 [95% CI 0.74−1.15]; p = 0.47) or in sensitivity analyses (Table S4), with variable between cluster primary outcome rates (ICC 0.013), and without differences in the components of the primary outcome (Table 2).

In intervention (vs. control) arms, there was no difference in birth preparedness or complication readiness (71.3% vs. 84.5%, respectively; aOR 0.65 [99% CI 0.03, 14.4]; p = 0.72).

There were no differences in other secondary outcomes between trial arms (Table 3). Miscarriage (8%), pregnancy termination (≈5%) and preterm birth (≈13%) complicated a minority of pregnancies, with very few home births (3.6% intervention vs. 3.9% control). There was no difference in comprehensive emergency obstetric care facility deliveries. Most women had spontaneous vaginal deliveries (Caesarean deliveries: ≈20%).

**Table 3:** Secondary, safety, and other outcomes.

	Intervention (n = 7839 pregnancies)	Control (n = 6944 pregnancies)	Adjusted OR^a^ (99% CI)	p-value
**Secondary outcomes**				
Birth preparedness and complication readiness^b^ (n (%))	5587 (71.3%)	5869 (84.5%)	0.65 (0.03, 14.4)	0.717
Proportion of facility births (n (%))	6073 (76.4%)	5334 (77.5%)	1.06 (0.92, 1.21)	0.302
Birth at a CEmOC facility (n (%))	3949 (65.5%)	3645 (68.3%)	0.9 (0.60, 1.34)	0.482
**Other outcomes**				
**Safety outcomes**				
SAEs unrelated to intervention (n (%))	0 (0%)	0 (0%)	NA	NA
Adverse events				
Transport-related injury or death (n (%))	0/401 (0%)	NA	NA	NA
Injection site haematoma/infection after community administration of IM MgSO_4_ (n (%))	0/47 (0%)	NA	NA	NA
Injection site complications after any administration of IM MgSO_4_ (n (%))	4/168 (2.4%)	NA	NA	NA
Respiratory depression, coma or death during transport following in-community MgSO_4_ (n (%))	0/47 (0%)	NA	NA	NA
Maternal sBP < 110 mmHg on facility arrival following in-community methyldopa	1/51 (2.0%)	NA	NA	NA
Deliveries (all pregnancy outcomes (total n)	6908	6109	**–**	**–**
Miscarriage (n (%))	553 (8.0%)	460 (7.5%)	1.20 (0.79, 1.281)	0.259
Medically terminated pregnancies (n (%))	319 (4.6%)	310 (5.1%)	0.94 (0.66, 1.35)	0.682
Live birth (n (%))	5842 (74.5%)	5182 (74.6%)	0.94 (0.80, 1.10)	0.291
Gestational age at delivery (week) (median (IQR))	39.1 (37.0–40.4)	39.3 (37.1–40.4)	–	0.032
Deliveries < 37 weeks (n (%))	793 (13.1%)	643 (12.0%)	1.07 (0.91, 1.27)	0·265
Deliveries < 34 weeks (n (%))	304 (5.0%)	236 (4.4%)	1.13 (0.79, 1.62)	0.377
*Missing (n (%))*	10 (0.1%)	2 (0%)	**–**	**–**
Mode of delivery (excluding miscarriage and terminations)			1.06 (0.92, 1.21)	0.302
Spontaneous vaginal (n (%))	4670 (77.4%)	4066 (76.2%)	**–**	**–**
Assisted vaginal (n (%))	66 (1.1%)	39 (0.7%)	**–**	**–**
Caesarean (n (%))	1297 (21.5%)	1235 (23.1%)	**–**	**–**

Data presented as median (interquartile range) or number (%) or number only.

OR = odds ratio. CEmOC = Comprehensive emergency obstetric care. IQR = interquartile range. sBP = systolic blood pressure.

a Odds ratio adjusted for individual-level (i.e., maternal age, parity, maternal primary education, previous delivery locations, and husband’s primary education) and cluster-level (i.e., baseline neonatal mortality rate and population density) characteristics.

b Birth preparedness and complication readiness was defined as an answer to ALL three of the following: 1) arranged for transport, 2) obtained prior permission to seek emergency care, and 3) saved money for obstetric care.

There was an apparent, but complex, relationship between the intensity of POM-guided contacts per pregnancy and outcomes in intervention arm women (Table 4 and Fig. S2). Compared with women without POM-guided contacts, women with 4–7 contacts had more adverse fetal and neonatal events (aOR 1.42 [95% CI 1.05, 1.93]; p = 0.025), based upon more neonatal morbidity (aOR 1·56 [95% CI 1.10, 2.22]; p = 0.012). Women who received ≥8 POM-guided contacts, suffered fewer stillbirths (aOR 0.19 [95% CI 0.10, 0.35]; p < 0.001), probably at the expense of more neonatal morbidity (aOR 1.39 [95% CI 0.97, 1.99]; p = 0.072).

**Table 4:** Relationship between intensity of POM-guided CLIP contacts and the primary outcome.

Outcomes	Number of POM-guided visits										
	0 visits		1–3 visits			4–7 visits			≥8 visits		
	Event rate	Adjusted OR (95% CI)^b^	Event rate	Adjusted OR (95% CI)^b^	p	Event rate	Adjusted OR (95% CI)^b^	p	Event rate	Adjusted OR (95% CI)^b^	p
Primary outcome^c^	93 (18.3%)	*Reference*	196 (23.1%)	1.22 (0.92, 1.63)	0.17	280 (23.7%)	1.31 (0.99, 1.73)	0.06	671 (19.2%)	0.94 (0.71, 1.26)	0.69)
Maternal outcome	31 (6.1%)	*Reference*	60 (7.1%)	1.16 (0.73, 1.85)	0.52	88 (7.4%)	1.30 (0.83, 2.04)	0.25	181 (5.2%)	1.00 (0.62, 1.59)	0.98
*Maternal mortality*	0 (0.0%)	*Reference*	0 (0.0%)	inestimable	–	0 (0.0%)	inestimable	–	4 (0.1%)	inestimable	–
*Maternal morbidity*	31 (6.1%)	*Reference*	60 (7.1%)	1.16 (0.73, 1.85)	0.52	88 (7.4%)	1.30 (0.83, 2.04)	0.25	180 (5.2%)	0.99 (0.62, 1.59)	0.98
Fetal or neonatal adverse outcome	72 (14.2%)	*Reference*	155 (18.2%)	1.23 (0.90, 1.70)	0.20	239 (20.2%)	1.42 (1.05, 1.93)	0.025	544 (15.6%)	0.91 (0.66, 1.99)	0.55
*Stillbirth*	20 (3.9%)	*Reference*	46 (5.4%)	1.20 (0.69, 2.10)	0.52	61 (5.2%)	0.90 (0.52, 1.55)	0.70	64 (1.8%)	0.19 (0.10, 0.35)	<0.001
*Neonatal mortality*	18 (3.7%)	*Reference*	36 (4.5%)	1.48 (0.78, 2.80)	0.23	42 (3.7%)	1.30 (0.69, 2.45)	0.42	83 (2.4%)	0.79 (0.41, 1.53)	0.48
*Neonatal morbidity*	51 (10.0%)	*Reference*	105 (12.4%)	1.16 (0.80, 1.68)	0.44	177 (15.0%)	1.56 (1.10, 2.22)	0.012	480 (13.8%)	1.39 (0.97, 1.99)	0.072

CI, confidence interval; OR, odds ratio.

^a^These analyses included the women in intervention clusters who were followed-up, excluding the ## who were recruited and had pregnancy loss prior to 20 weeks.

b Adjusted for maternal characteristics (as in the primary analysis – maternal age, parity, and basic education; enrolment timing in the trial; and distance from the household to facility.

c Defined as one/more of maternal morbidity or mortality, stillbirth, neonatal mortality, or neonatal morbidity. This was the primary outcome in the CLIP Trials.

No adverse events were reported overall or related specifically to administration of oral methyldopa or intramuscular MgSO_4_ in the community by any cadre of worker.

## 4 Discussion

In Northwest Karnataka, the CLIP intervention had no significant impact on maternal, fetal, or newborn mortality or major morbidity, in the primary or any sensitivity analyses. This was despite all components of the CLIP intervention being evidence-based.

It is probable that we were underpowered to find the pre-specified difference in maternal and perinatal mortality and morbidity; however, this is unlikely to have influenced the outcome of the trial. While over 14,000 women were recruited, the small number of clusters (N = 12) on which statistical power is based, had outcome rates that were more different than anticipated (i.e., ICC 0.013), rather than the 0.001 anticipated from previous regional cluster RCT experience using the MNH Registry [23].

Maternal health in rural Karnataka is a construct of various determinants including low autonomy of women, limited transportation, financial constraints, and incentive-based programmes [7]. A limitation is poor socioeconomic profiling, as health outcomes were probably worse among women belonging to scheduled castes, scheduled tribes, and the impoverished [7]. However, adjusting for level of education (both maternal and paternal) did not reveal a difference between arms.

This is one of the largest prospective studies in India and the MNH Registry surveillance and follow-up procedures ensured complete follow-up, as observed previously [15], [21]. Women were generally enrolled at quite an early gestational age, which facilitated reporting of reliable event rates for miscarriage (≈8%) and elective termination (≈5%) in these communities. In addition, we assessed population-level estimates of maternal, fetal, and neonatal mortality and major morbidity that were not subject to recall bias.

The complex intervention was implemented well, but, by design, only at community level. A total of 1379 community engagement sessions were held and 148 CHWs trained to deliver the intervention. Seventy-six percent of enrolled women received ≥1 POM contact; this is consistent with regional experience [26]. While only 42% of intervention arm women received the planned frequency of contacts, we observed that receipt of ≥8 POM contacts reduced the burden of stillbirth, at the cost of survivable neonatal morbidity apparent with intensity of ≥4 contacts per pregnancy. An important justification for the WHO eight-contact model of antenatal care has been the reduction of stillbirth [15]. These data support that conjecture.

With special government permission, we engaged existing healthcare workers and strengthened evidence that ASHAs and Anganwadi workers are capable of task-sharing, including community measurement of BP and proteinuria testing [27]. These workers were able to observe women for emergency conditions, assess symptoms and signs of pre-eclampsia, enter findings into the POM app, and operationalise POM recommendations for triage, transport, and treatment (by engaging with ANMs and staff nurses). ANMs and staff nurses were able to safely administer oral methyldopa and intramuscular MgSO_4_, in their communities, as authorised by national guidelines. This should encourage others to address implementation gaps for treatment guidelines, particularly related to MgSO_4_ administration [9].

Other cultural norms whereby women in both arms migrated to their mothers' homes may have confounded the relation between intervention and outcomes, as women may have received a lower frequency of antenatal and postnatal CLIP contacts. External policy factors such as the provision of the postnatal *Madilu* kits and *Janani Suraksha Yojna* could have influenced care-seeking among trial participants [12]. In addition, regular data monitoring procedures shared by health care providers from all clusters may have obscured any direct effect of the intervention.

Notwithstanding the careful design of the intervention, we based our threshold for treatment and referral on systolic hypertension to increase generalisability where functioning sphygmomanometers and training are lacking. While the incidence of isolated diastolic hypertension was 4.8% in our study population, this was unlikely to have affected the impact of the CLIP intervention given the overall lack of treatment signal on mortality and morbidity for the mother and baby [3].

Another important limitation is that the CLIP intervention was focussed on increasing responsiveness to health care within communities (including PHCs), and not optimising facility-based care. Therefore, outcomes may have been largely determined by secondary or tertiary facility care; these facilities were common to both arms. Standardising facility care improves maternal outcomes related to pregnancy hypertension [5], [6], and could improve upon the minimal impact of National Rural Health Mission-related increased institutional deliveries on national mortality rates [12]. Karnatakan facilities are constrained by human resource shortages, inconsistent power, inadequate cleanliness, weak information systems, poor referral chains, and poor governance and accountability measures [9], [12]. Regional MgSO_4_ use is hampered by stock-outs, training gaps, and non-standardised formulations and regimens [13].

Care-seeking in CLIP differed from District Level Health Survey-4 (DLHS-4), in terms of any ANC from a skilled provider (>99% [CLIP] vs 88% [DLHS-4]), ≥4 ANC visits (76% [CLIP] vs 70% [DLHS-4]), and rates of home birth (<4% [CLIP] vs 4% for delivery at home [DLHS-4]) [28]; implying that data collection sensitised women in all clusters to the importance of birth preparedness and complication-readiness.

Most women (almost 75%) booked before 20 weeks, and many suffered a miscarriage or elective termination of pregnancy (12.6% in both intervention and control clusters). Preterm birth rates of 13.1% and 12.0% in intervention and control clusters, respectively; are consistent with those from other trials in Karnataka (e.g., 14%) [29].

Our population estimates of mortality and major morbidity differ variably from those previously published. We observed a maternal mortality ratio of 136 per 100,000 livebirths [95% CI 90, 230], which is consistent with data from 2014 to 6 state-level government (108) [30], Haryana (191) [31], and national-level sources (207) [1], but considerably lower than data from Uttar Pradesh (399) [31].

The CLIP stillbirth rate (per 1000 livebirths) (31.5) is similar to that recorded in our baseline study (28.6) and Uttar Pradesh (37.6) [15], [31], but higher than Haryana (22.3) and facility-based studies in Karnataka (12.0) [31], [32], possibly reflecting better ascertainment. The neonatal mortality rate estimate (per 1000 livebirths) was 24.4, which is lower than estimates from Haryana (40.0) and Uttar Pradesh (41.7) [31]. There are no comparable published estimates for miscarriage or elective termination rates.

Previously-published estimates of major maternal and neonatal morbidity are limited. Blood transfusion was the most-common maternal morbidity (3.2%) and was twice our baseline study rate [15], and may reflect the higher prevalence of anaemia in all clusters (84%) than previously reported in either Belagavi (44%) or Bagalkote (56%) [33]. We systematically measured haemoglobin in all enrolled women.

Our incidence of sepsis (broad definition of fever and symptoms, incidence: 0.9%) is similar to our baseline study (same definition, 0.8%); both of these estimates are lower than the clinically-detected incidence of puerperal sepsis following home (1.4%) and facility (1.2%) deliveries in a community-based study of 4975 rural Rajasthani women.[34] The CLIP rate of antepartum haemorrhage (0.7%) is lower than that reported in a prospective tertiary Mumbai facility-based study (1.3%), but similar to baseline [15]. The incidence of neonatal morbidities was higher in CLIP (vs. baseline study) [15] for breathing difficulty (6.6% vs. 4.1%), lethargy (5.1% vs. 2.8%), feeding difficulty (5.0% vs. 2.0%) as the MNH Registry matured and overcame under-reporting of outcomes due to a possible social desirability bias. In addition, the baseline study may have sensitised communities to identify, understand and report outcomes in the trial period [15], [35], resulting in a higher incidence recorded in the trial [34].

In conclusion, community-level interventions addressing triage, initial treatment, and transport of women with pregnancy hypertension can be successfully performed by ASHAs and ANMs, but their numbers must be adequate to provide at least eight ANC contacts to reduce adverse outcomes. Even then, the reduction is not large, suggesting that a community-only focus without facility enhancement is unlikely to yield improvements in maternal and perinatal outcomes. Further study should focus on community BP measurement and simple condition-specific interventions, as part of a comprehensive health-strengthening programme. Finally, our prospectively gathered, population-level estimates of maternal, fetal and neonatal outcomes inform India’s journey to achieve universal health coverage using effective investment of public funds.

## Declaration of Interests

We declare no competing interests. BAP, LAM and PvD acknowledge that the intellectual property related to the miniPIERS prediction model used in the CLIP trials was transferred in its entirety from the University of British Columbia to them, among other inventors, prior to the trial. They have no financial benefit from the use of the model based on the transfer.

## Figures and Tables

**Supplementary figure 2:**