# Supplementary material for: Community level interventions for pre-eclampsia (CLIP) in India: A cluster randomised controlled trial
Source: Pregnancy Hypertens. 2020 Jul;21:166–75. doi: 10.1016/j.preghy.2020.05.008 (PMC7471838; doi:10.1016/j.preghy.2020.05.008)
Supplement: Supplementary data 1 [file mmc1.docx]

# **CLIP Trials Data Sharing Statement**

The CLIP Trial data are de-identified participant-level data. Once the primary CLIP manuscripts, individual participant data meta-analysis, and papers based on the other pre-defined analyses are published as per the Statistical Analysis Plan (SAP), the data will be freely available to academically-active entities (e.g., universities, NGOs, multilaterals), with the CLIP Principal Investigator (Peter von Dadelszen) or named delegate as a named co-investigator, for the purposes of pregnancy-related research and within the limits of the informed consent obtained. Access will be through the CLIP Trials Data Access Committee*, contacted at ‘PRE-EMPT@cw.bc.ca’, as referenced on our website at ‘https://PRE-EMPT.bcchr.ca’. A full data dictionary and all study documents will be available. Access will be through written application. When approved, a quote for the costs of preparing the data will be provided to the applicant.

By submitting an application form, the investigator agrees that s/he has read, understood and agrees to the terms and conditions below:

1. S/he is an academically-active researcher affiliated with an entity able to engage in a data transfer agreement;
2. S/he warrants that the information entered is to the best of her/his knowledge full and correct;
3. S/he agrees that the Data Sharing Agreement will only be used for the specific project outlined in the application;
4. S/he represents that s/he has obtained the necessary approvals to transfer the data and/or receive the data under this Data Sharing Agreement;
5. S/he understands that the responses provided will form part of a legally-binding document;
6. S/he understands that the Agreement is not valid until a fully-executed copy, with signatures from all parties, is emailed to PRE-EMPT@cw.bc.ca); and
7. S/he understands that no modifications can be made to the Data Sharing Agreement and if modifications are made, the Data Sharing Agreement will be rendered invalid.

There is no pregnancy-specific repository for us to access, but once the primary papers for the CLIP Trials have been published, we will be depositing a copy of our data in the HBGDki repository at the Bill & Melinda Gates Foundation, our funder. The permitted uses and disclosures of these data are as follows:

1. The Foundation will limit the use and disclosure of the CLIP data to conduct research related to achieving the goals of the Foundation as represented above. The Foundation may also de-identify the data set and aggregate it with other de-identified information.
2. The Foundation will restrict access to the CLIP data to individuals involved in the Foundation’s research who have a need to access the CLIP data to carry out their duties as they relate to the Permitted Uses and Disclosures identified above, and any such access will be consistent with the assurances and obligations set forth in this Agreement. The Foundation will use appropriate safeguards to prevent use or disclosure of the CLIP data other than as permitted by this Agreement.
3. The Foundation will report to HBGDki Collaborator any use or disclosure of the CLIP data not provided for by this Agreement of which the Foundation becomes aware.
4. The Foundation will ensure that any agents, including subcontractors, to whom it provides the CLIP data, if any, agree to the same restrictions and conditions that apply to the Foundation with respect to such information.

** The* ***Data Access Committee*** *is made up of the following individuals: Peter von Dadelszen and Laura A. Magee (King’s College London, UK); Zulfiqar A Bhutta (Aga Khan University, Karachi, Pakistan and the Hospital for Sick Children, Toronto, Canada); Rahat N Qureshi (Aga Khan University, Karachi, Pakistan); Ashalata A Mallapur (S Nijalingappa Medical College, Bagalkote, India); Mrutyunjaya B Bellad and Shivaprasad Goudar (KLE Academy of Higher Education and Research’s JN Medical College, Belagavi, India); Khátia Munguambe, Charfudin Sacoor, and Esperança Sevene (Centro de Investigação em Saúde da Manhiça, Manhiça, Mozambique)*
