# Supplementary material for: Community level interventions for pre-eclampsia (CLIP) in India: A cluster randomised controlled trial
Source: Pregnancy Hypertens. 2020 Jul;21:166–75. doi: 10.1016/j.preghy.2020.05.008 (PMC7471838; doi:10.1016/j.preghy.2020.05.008)
Supplement: Supplementary data 4 [file mmc4.pdf]

Data Safety and Monitoring Board Charter for the CLIP (Community Level Interventions for Pre-eclampsia) Cluster Randomized Controlled Trial

1. Introduction

This Charter is for the Data and Safety Monitoring Board (DSMB) for the CLIP (Community Level Interventions for Pre-eclampsia) cluster randomized controlled trial.

The Charter is intended to be a living document. The DSMB may wish to review it at regular intervals to determine whether any changes in procedure are needed.

2. Responsibilities of the DSMB

The role of the DSMB will be to deal with any ethical issues that may arise while the trial is in progress and at the planned interim analyses (½ planned sample size). Serious adverse events (SAEs) will be reported to the DSMB as outlined below. SAEs should be: (i) serious, (ii) unexpected (in nature, severity, or frequency), and (iii) thought to be related to the study intervention. This plan reflects the expected nature of the other AE, which are events of note but that do not necessitate stoppage of the trial due to safety concerns prior to the interim analysis.

SAE reporting

- When all required information has been gathered in the reporting field site, the field site supervisor will forward 'blocked' copies (without patient, site or allocated intervention group identifiers) of all documentation and an updated 'Serious Adverse Event Form' to the National CLIP Trial Co-ordinating Centre. That centre will assess whether or not: (i) additional information is required, and (ii) the National Principal Investigator and Co-ordinator should be informed immediately, or at the next scheduled meeting. *The **documents to be reviewed** are: (i) all data forms (including those derived from the POM tool) for each women with a reported primary outcome (for mother or baby), masked to the cluster allocation, and (ii) copies of the relevant facility documents (if applicable) that serve as the 'source documents' and detail the outcome for the woman/baby. If the outcome were reported in the community with no associated facility admission, then the data forms should be reviewed for consistency and to ensure that the woman's story makes sense from a clinical perspective.*
- After review (and collection of additional information, as applicable) of a reported SAE by the relevant National CLIP PI and National CLIP Trial Co-ordinating Centre, the 'blocked' documentation (including the SAE Form and summary of the case) **should be reviewed by the in-country National Outcomes Adjudication Committee (Appendix I). That Committee will decide whether or not the reported SAE is a true SAE or a pre-specified Trial outcome. Then**, the 'blocked' documentation (including the SAE Form and summary of the case) and the Committee's opinion should be sent to the UBC Trial Co-ordinating Centre (TCC) for review by the UBC Outcomes Adjudication Committee; if UBC agrees that the reported SAE is a true SAE, UBC will send the report to the DSMB for urgent review. However, if UBC agrees that the reported SAE is a pre-specified Trial outcome, then the report will be sent to the DSMB at the time of the next analysis.
- The UBC Outcomes Adjudication Committee decision will be reviewed by the CLIP Trial Steering Committee (SC) at the next scheduled quarterly teleconference, or earlier if the UBC Outcomes Adjudication Committee feels that this is appropriate. Possible reasons for urgent review by the SC are because the reported SAE (i) is not a pre-specified trial outcome (i.e. it is deemed to be a true SAE), or

because (ii) is a pre-specified trial outcome but the UBC Outcomes Adjudication Committee has further concerns. After review of the SAE by these groups, the UBC TCC will generate and distribute a summary report of the Serious Unexpected Event(s) to be reported and reviewed by the DSMB at the time of the interim analyses, or earlier if either the UBC Outcomes Adjudication Committee and/or SC have additional concerns.

- The summary report should include the 'Serious Unexpected Event Form' received from the site, and a typed summary of all accumulated supporting documentation for each reported event. Ensure all patient, site or allocated intervention group identifiers have been removed. The DSMB can then request further information if they wish to take a closer look at any particular event.
- For reported events ruled by the Outcomes Adjudication Committee to be pre-specified trial outcomes (and NOT true SAEs), the UBC TCC will: (i) communicate with the reporting site to let them know about the outcome of the evaluation of their reported Serious Unexpected Event by the UBC Outcomes Adjudication Committee; (ii) inform them that the reported event will also be reviewed by the SC and included in a summary report made to the DSMB at the time of the next scheduled analysis (after DSMB review, a written summary of all reported Serious Unexpected Events, and the DSMB's ultimate assessment of these events, will be distributed to their site (and all sites)); and (iii) invite the site to inform the TCC if they have any concerns.
- For reported events ruled by the UBC Outcomes Adjudication Committee and SC to be true SAEs, UBC TCC will: (i) communicate with the reporting site to let them know about the outcome of the evaluation of their reported Serious Unexpected Event by the UBC Outcomes Adjudication Committee and Steering Committee; (ii) inform them that the reported event will also be included in a summary report made to the DSMB (either immediately or at the time of the next scheduled analysis, as decided by the Steering Committee) (after DSMB review, a written summary of all reported Serious Unexpected Events, and the DSMB's ultimate assessment of these events, will be distributed to their site (and all sites)); and (iii) invite the site to inform the UBC TCC if they have any concerns.
- For all reported events, following review by the DSMB, the Principal Investigator will prepare a written summary of the reported Serious Unexpected Event(s), and the DSMB's assessment of these event(s). If the event(s) is (are) deemed to be a true SAE, the anonymised SAE Report Form(s) will also be attached. This report will be submitted to: (i) UBC REB. As the Clinical and Data Co-ordinating Centre, UBC will inform the UBC REB of all reported Serious Unexpected Events; (ii) the study sponsor, BMGF, will be informed through the routine monthly/alternate monthly dialogue; and (iii) all Site Investigators, including the reporting Investigator. Each Site Investigator will be responsible for reviewing this Serious Unexpected Event summary report and forwarding it to their local REB. It will be recommended that each Site Investigator check with their local REB for other local reporting requirements.

3. DSMB Membership

- **Professor Eileen Hutton (Chair)**

  Associate Professor, Department of Obstetrics and Gynaecology, Assistant Dean, Faculty Health Sciences, and Director, Midwifery Education Program, McMaster University, Hamilton, ON, Canada (http://fhs.mcmaster.ca/ceb/faculty_member_hutton.htm).

- **Lehana Thabane**

  Professor and Associate Chair, Department of Clinical Epidemiology & Biostatistics, Director, Biostatistics Unit, Centre for Evaluation of Medicine, and Senior Scientist, Population Health Research Institute (PHRI), Hamilton Health Sciences, McMaster University

  (http://fhs.mcmaster.ca/ceb/faculty_member_thabane.htm).

- **Romano Byaruhanga**

  Consultant Obstetrician, Nsambya, Uganda and President of the Association of Obstetricians and Gynaecologists of Uganda.

  (http://www.sogc.org/aogu/index.aspx?contentID=41).

- **Mario Merialdi**

  Senior Director, Maternal and Newborn Health, Global Health, Becton, Dickinson and Company
  email: mario_merialdi@europe.bd.com

- **Brian A. Darlow**

  CureKids Professor of Paediatric Research, University of Otago Christchurch, PO Box 4345
  Christchurch 8140, New Zealand

  (http://www.otago.ac.nz/healthsciences/expertise/profile/index.html?id=229)

4. Responsibilities of the CLIP Trial Co-ordinating Centre At UBC

- The CLIP co-ordinating centre at UBC is responsible to the DSMB for the following:
  - Making resources available to the DSMB as required to carry out its designated functions.
  - Creating and maintaining statistical reports for the DSMB.

5. Responsibilities of the Data Management Centre

- The Data Management Centre will be responsible for the following:
  - Collection and on-site monitoring of case report forms (CRF).
  - Ensuring the completeness and accuracy of all data collected to the extent required by the DSMB.
  - Providing analysis data sets to the Statistician/Data Analysts containing all CRF data necessary for creating DSMB reports.

6. Scheduling, Timing, and Organization of Meetings

The DSMB will meet by teleconference held approximately twice a year, with additional meetings or conference calls scheduled as needed. Meetings and conference calls will be scheduled by the CLIP Trial Co-ordinating Centre.

The agenda for DSMB meetings and calls may be drafted by the CLIP Trial Co-ordinating Centre. A member of CLIP Trial Co-ordinating Centre will finalize the agenda after consultation with the DSMB Chair. The agenda and meeting materials should be distributed by the CLIP Trial Co-ordinating Centre at least one week before each meeting or call.

The Steering Committee statistician is on the call for the first few minutes to answer questions regarding variable definitions and table structures and is available to the DSMB by phone to answer further questions. Prior to the call the DSMB chair and members are provided with a copy of the study protocol, the latest recruitment table, and a patient information sheet (useful to non-clinician members). The DSMB may wish to discuss at the first or subsequent meetings what data they wish to review and how it should be presented. The DSMB can also review adverse event data, other safety data, quality and completeness of study data, and enrollment data at each meeting to ensure proper trial conduct. At intervals, as noted above, the DSMB will also review formal interim analyses of the primary end point.

Before each meeting, when the agenda is sent out, a member of CLIP Trial Co-ordinating Centre will ask all DSMB members to state whether they have developed any new conflicts of interest since the last formal meeting. If a new conflict is reported, the Chair and CLIP Trial Co-ordinating Centre will determine if the conflict limits the ability of the DSMB member to participate in the discussion.

It is expected that all DSMB members will attend every meeting and call. However, it is recognized that this may not always be possible. Therefore, the DSMB may wish to discuss whether establishing a quorum for voting is desirable. All standing Monitoring Board members are voting members. The Board may also wish to decide in advance whether *ad hoc* members can vote.

7. Meeting Format

The DSMB usually meets by teleconference. The Steering Committee statistician is on the call for the first few minutes to answer questions regarding variable definitions and table structures and is available to the DSMB by phone to answer further questions. Prior to the call the DSMB chair and members are provided with a copy of the study protocol, the latest recruitment table, and a patient information sheet (useful to non-clinician members).

Appendix I

Protocol for adverse event reporting and outcome adjudication in CLIP

This document contains a description of the expected reporting timelines and process for both the primary outcome and serious adverse events (SAEs) or adverse events that occur during the CLIP Pilot and Definitive trials. A summary table is provided on page 8 of this document listing all types of reportable events and the levels of review each requires.

Part 1: Outcomes Adjudication

Within each country, all incidents of the primary outcome must be reviewed and confirmed by the National Outcomes Adjudication Committee. Step-by-step instructions for the outcome review are as follows:

1. Field staff will notify the site's trial coordinator regarding any occurrence of the primary outcome documented during regular surveillance.
2. All data collection forms required for review of the outcome are completed and entered into the study database.
3. After the data check is complete and any potential errors in data entry are corrected for the month in which the outcome occurred, a member of the data management team will print copies of de-identified data forms relating to each case to be reviewed. The relevant forms to be reviewed are the following:
    a. pregnancy registration
    b. regular surveillance
    c. verbal autopsy (if applicable).

    NOTE: Facility surveillance form and those from the POM tool (for mother or baby) should NOT be reviewed, as our primary outcome will be based on community-level data only. (Centrally, at UBC, we will examine how the community-level data relate to the facility-level data.)
4. All forms printed are reviewed to ensure all personal (and cluster) identifiers have been removed and also to black-out any information that may identify the cluster allocation of the case.
5. A set of forms for each case to be reviewed is provided to each member of the **National Outcomes Adjudication Committee** prior to the monthly meeting. Each committee member should:
    a. Review the forms for consistency and ensure that the woman's story make sense from a clinical perspective.
6. *Prior to* the monthly meeting, each member of the committee reviews the case and documents (on a standardized **Outcomes Adjudication Form**), their

decision as to whether the outcome occurred and meets criteria for a primary outcome.

   a. If the mother or baby(ies) died, then the outcome to be adjudicated is death. The Committee does not need to adjudicate the preceding morbidity(ies).
   b. If the mother and/or baby(ies) suffered a morbidity(ies), then the Committee should adjudicate whether or not one/more morbidities occurred for EACH mother and EACH baby; each individual maternal or neonatal morbidity does not need to be adjudicated separately but all source data for all events will be reviewed.
   c. If there is uncertainty within the committee as to the occurrence of an individual mortality or morbidity event for a given woman or baby after review of the case files, this should initiate a data query to resolve any relevant questions or potential data errors. If multiple morbidities occurred and it is clear at least one is valid for a given woman or baby, this case can be adjudicated as having met criteria for the primary outcome even if data queries remain outstanding at the time of the committee meeting.
   d. If there is both a maternal event and a perinatal/neonatal event, then EACH of the maternal and perinatal/neonatal events should be adjudicated for completeness, even though only one of the events (perinatal and/or maternal) needs to be confirmed for the woman to be recorded as having had the primary outcome (as the woman is the unit of analysis in the trial).

7. During the monthly meeting, all members submit their decisions. Discussion will be necessary about any case for which two or more members disagree on the decision as to whether to include the event as a primary outcome; discussion should proceed until consensus is reached or a decision is made about additional data that may be required. Discussion should also occur about any case for which data appear to be inconsistent or have errors.
8. Final decisions on all cases are submitted to UBC on a monthly basis, using the standard reporting template (**Outcomes Adjudication Reporting Template**).

NOTE: The **National Outcomes Adjudication Committee** will consist of an uneven number of members of the national CLIP Team, including but not limited to one obstetrician, one paediatrician, and one methodologist/trialist. For review of an individual case, these individuals must not have been involved in the care of the woman being discussed. For example, if the obstetrician in the committee had consulted on a case where the woman had a suspected PPH, when that individual case is reviewed, the obstetrician cannot vote on it’s inclusion as a primary outcome.

Part 2: Serious Adverse Events (SAE) and adverse events

Serious adverse events that occur during the CLIP Pilot or Definitive trial are REPORTABLE to both the National Outcomes Adjudication Committee and, after review by that committee, to UBC who will also report to the DSMB. The timeframe for reporting to UBC and the DSMB is described in Table 1.

The term '**serious adverse event (SAE)** is to be used to describe events that are ALL of the following:

(i) serious
(ii) unexpected (in nature, severity, or frequency), and
(iii) thought to be related to the study intervention.

Review of these events will follow the same protocol as incidents of the primary outcome (described above) but a different SAE form will be used. However, if the event is also a pre-specified primary outcome of the trial, then a separate Outcomes Adjudication Form will be provided to the Outcomes Adjudication Committee for completion, in addition to the **SAE Form**.

Please NOTE that when the SAE information is provided to the DSMB, additional documentation will be included in the form of the (i) facility surveillance data and (ii) POM data (if applicable).

Other adverse events are NOT reportable to UBC except through the regular surveillance and POM data collection system but will be examined at the time of the interim analysis.
These are:

(i) For women who received **methyldopa** administration in the community: relative maternal hypotension on arrival at facility (defined as sBP<110 mmHg)
(ii) For women who received **MgSO4** in the community: either respiratory depression, coma or death during transport, as diagnosed upon arrival at facility
(iii) For women who were transported to facility: **Transport-related injury** (life or limb) **or death** during transport
(iv) For women who received $MgSO_4$ in the community or facility: Injection site haematoma or infection
(v) For women who were referred to facility: ≥ 20% of women being sent back to their communities without follow-up

Table 1a: Outcomes in the CLIP Trial

	Reportable (within ≤30d) to site's **National Co-ordinating Centre**	Review by **National Outcomes Adjudication Committee** on a regular basis* (without knowledge of cluster allocation)	Review by **UBC Trial Co-ordinating Centre (TCC) & Outcomes Adjudication Committee**	Review by the **CLIP Steering Committee**	Review by **DSMB**
**Maternal and perinatal mortality and morbidity‡** (p3)	**n/a**	✓ (regularly scheduled)	✓ (regularly scheduled)	**n/a**	✓ (regularly scheduled)

Table 1b: Adverse events in the CLIP Trial

	Reportable (within ≤30d) to site's **National Co-ordinating Centre**	Review by **National Outcomes Adjudication Committee** on a regular basis* (without knowledge of cluster allocation)	Review by **UBC Trial Co-ordinating Centre (TCC) & Outcomes Adjudication Committee**	Review by the **CLIP Steering Committee**	Review by **DSMB**
**Serious adverse events** (SAEs) †	✓	✓ (as soon as possible)	✓ (regularly or earlier if deemed to be appropriate by National Outcomes Adjudication Committee)	✓ (regularly or earlier if deemed to be appropriate by UBC TCC and Outcomes Adjudication Committee)	✓ (regularly or earlier if deemed to be appropriate by CLIP Steering Committee)
**Adverse events**	**n/a**	**n/a**	✓ (regularly scheduled)	**n/a**	✓ (regularly scheduled)

POM Tool (Piers On the Move)

*** *Review of these SAEs may require scheduling of additional ad hoc meetings.***

† Many of these reported will be pre-specified trial outcomes. If so according to the National Outcomes Adjudication Committee, these events will be reviewed by the DSMB.

‡ MATERNAL AND PERINATAL MORTALITY AND MORBIDITY

Maternal death

Maternal morbidity - These are the serious end-organ complications of pre-eclampsia, other major causes of maternal mortality, or life-saving interventions related to one of the aforementioned:

Serious end-organ complications of pre-eclampsia:

- *Eclampsia:* occurrence of generalised convulsions during pregnancy, labour or within 42 days of delivery in the absence of epilepsy or another condition predisposing to convulsions
- *Stroke:* hemiparesis and/or blindness developed during pregnancy or in the 42 days postpartum lasting greater than 48 hours
- *Coma:* prolonged unconsciousness ≥12 hours
- *Antepartum haemorrhage:* vaginal bleeding ≥ 15 mL with or without pain before the onset of labour
- *Disseminated intravascular coagulation* (DIC): abnormal bleeding from mucosa (mouth and/or ears)

Other major causes of maternal mortality:

- *Obstetric sepsis:* In the community, defined as fever and one of: abdominal/uterine tenderness, foul smelling vaginal discharge/lochia, productive cough and shortness of breath, dysuria or flank pain, headache and neck stiffness. In the facility, defined as presence of fever (>38°C), a confirmed or suspected infection (e.g., chorioamnionitis, septic abortion, endometritis, pneumonia) and at least one of the following: heart rate >90/min, respiratory rate >20/min, leukopoenia (total leukocyte count [TLC] <4 x 10^9/L) or leukocytosis (TLC >12 x 10^9/L)
- *Vesicovaginal or rectovaginal fistula:* continuous loss of urine and/or faeces after delivery

Life-saving interventions:

- *Cardiopulmonary resuscitation:* a set of emergency procedures including chest compressions and lung ventilation applied in cardiac arrest victims
- *Dialysis:* haemodialysis and/or peritoneal dialysis
- *Mechanical ventilation* (other than for Caesarean section): intubation and ventilation not related to anaesthesia
- *Blood transfusion:* ≥1 unit
- *Interventions for major postpartum haemorrhage:* brace sutures, external and internal uterine compression, anti-shock garment use, internal iliac artery ligation and/or hysterectomy with or without transfusion

Perinatal & late neonatal death - defined as stillbirth [≥20^{+0} and/or ≥500g], early neonatal mortality [d 0-7 of postnatal life] and late neonatal mortality [d 8-28 of postnatal life] / 1,000 identified pregnancies

Neonatal morbidity - defined as non-lethal events of seizure and coma[30] during d 0-28 of postnatal life / 1,000 identified pregnancies): *feeding difficulty, breathing difficulty, seizure, lethargy, coma, fever, hypothermia, umbilical cord infection, skin infection, bleeding, jaundice, vomiting/diarrhoea*
