# Supplementary material for: Community level interventions for pre-eclampsia (CLIP) in India: A cluster randomised controlled trial
Source: Pregnancy Hypertens. 2020 Jul;21:166–75. doi: 10.1016/j.preghy.2020.05.008 (PMC7471838; doi:10.1016/j.preghy.2020.05.008)
Supplement: Supplementary data 5 [file mmc5.pdf]

CLIP INDIA Interim analysis

Date of report: May 17, 2016
Date of database lock: January 19, 2016

Description of the CLIP interim analysis

An interim analysis is planned for each of the three countries once complete data (until 42 days postpartum) has been received for women making up half of the planned sample size for that country. In the event that the site is unable to reach planned sample size, due to logistical and pragmatic challenges, then the interim analysis will be conducted once complete pregnancies (until 42 days postpartum) are *expected* for women making up half of the projected sample size for that country. Projection of sample size for each country will be first computed on March 2016, based on the observed recruitment rates in the Definitive trial as follows: 12 months of recruitment in Pakistan, 15 months of recruitment in India, and 11 months of recruitment in Mozambique. The average monthly recruitment will be calculated and applied to the remaining months of the trial, to obtain the final projected sample size.

The stopping rule for both benefit and harm will require an observed difference in the composite primary outcome rate between groups associated with an alpha <0.001.

	India
Planned sample size (births)	16800
Current sample size (births)	10467

Table 1. Baseline characteristics of patients

Characteristic	Group A	Group B
	5558	4909
**Patient-level data**		
Maternal age at enrollment (yr)	23.1+/-3.2 23 [20; 25]	23+/-3.1 23 [20; 25]
- ≤ 17 yr	11 (0.2%)	8 (0.2%)
- 18-34 yr	5495 (98.9%)	4869 (99.2%)
- ≥ 35 yr	52 (0.9%)	32 (0.7%)
Parity (%)		
- 0	1985 (35.7%)	1682 (34.3%)
- 1	2023 (36.4%)	1759 (35.8%)
- ≥ 2	1550 (27.9%)	1467 (29.9%)
Level of education		
Illiterate	768 (13.8%)	794 (16.2%)
Literate no school	209 (3.8%)	139 (2.8%)
School	4581 (82.4%)	3976 (81%)
Gestational age at enrollment (wk)	12.7+/-6.1 11 [8; 15]	13.9+/-6.8 13 [9; 17]
Method for ascertaining gestational age		

Last Menstrual Period	4127 (99.8%)	3579 (99.9%)
Clinical exam	56 (1.4%)	316 (8.8%)
Ultrasound	339 (8.2%)	297 (8.3%)
Past history		
Hypertension outside pregnancy	99 (1.8%)	64 (1.3%)
Hypertension in pregnancy	176 (4.7%)	174 (5.2%)
Care in last pregnancy		
Place of delivery		
Home	299 (8.1%)	284 (8.4%)
Sub-centre	10 (0.3%)	11 (0.3%)
Primary Health Centre	1482 (40%)	1010 (30%)
Government hospital	931 (25.1%)	1128 (33.5%)
Private clinic	115 (3.1%)	132 (3.9%)
Private hospital	866 (23.4%)	786 (23.4%)
On route to facility	2 (0.1%)	2 (0.1%)
Other	2 (0.1%)	7 (0.2%)
Don’t know	1 (0%)	3 (0.1%)

Table 2. Pregnancy outcomes and CLIP trial primary outcome

	Group A	Group B	
N participants	5558	4909	
N infants	5602	4937	
**Birth outcomes**			
Miscarriage – (N(N%))	382 (6.9%)	363 (7.4%)	
Medically Terminated Pregnancies – (N(N%))	260 (4.7%)	241 (4.9%)	
Stillbirth– (N(N%))	118 (2.1%)	91 (1.9%)	
Livebirth– (N(N%))	4801 (86.4%)	4217 (85.9%)	
Early neonatal death – (N(N%))	113 (2.4%)	75 (1.8%)	
Late neonatal death – (N(N%))	29 (0.6%)	28 (0.7%)	
**Neonatal outcomes (any of the following)**	762 (13.7%)	698 (14.2%)	
Stillbirth or neonatal deaths	258 (4.6%)	192 (3.9%)	
Neonatal Morbidity (any of the following)	508 (9.1%)	510 (10.4%)	
Breathing problems	339 (6.1%)	350 (7.1%)	
Feeding problems	249 (4.5%)	268 (5.5%)	
Lethargy	273 (4.9%)	234 (4.8%)	
Coma (neonatal)	35 (0.6%)	15 (0.3%)	
Seizure (neonatal)	44 (0.8%)	45 (0.9%)	
Umbilical stump infection	15 (0.3%)	30 (0.6%)	
Skin infection	83 (1.5%)	32 (0.7%)	
Jaundice	17 (0.3%)	37 (0.8%)	
Diarrhea/vomiting	103 (1.9%)	102 (2.1%)	
Bleeding	70 (1.3%)	57 (1.2%)	
**Maternal outcomes (any of the following)**	256 (4.6%)	222 (4.5%)	
Maternal death	0 (0.0%)	1 (0.0%)	
Maternal Morbidity (any of the following)	256 (4.6%)	222 (4.5%)	
Blood transfusion	171 (3.1%)	134 (2.7%)	
Hysterectomy	1 (0.0%)	2 (0.0%)	
Antepartum haemorrhage	55 (1.0%)	34 (0.7%)	
Coma	2 (0.0%)	2 (0.0%)	
Fever with symptom	69 (1.2%)	81 (1.7%)	
Received Anti-shock garment	0 (0.0%)	0 (0.0%)	
Received Bimanual uterine compression	0 (0.0%)	0 (0.0%)	
Received Brace sutures	0 (0.0%)	0 (0.0%)	
Received CPR	1 (0.0%)	0 (0.0%)	
Received Dialysis	0 (0.0%)	1 (0.0%)	
Received Internal iliac artery ligation/devascularisation procedure	0 (0.0%)	1 (0.0%)	
Received Mechanical ventilation	6 (0.1%)	0 (0.0%)	

Seizure (fits)	15 (0.3%)	12 (0.2%)	
Stroke	3 (0.1%)	2 (0.0%)	
**CLIP trial primary outcome** (Any of the list of Neonatal Outcomes or the list of Maternal outcomes presented above)	936 (16.8%)	847 (17.3%)	0.72*
**Population standardized difference** (95% confidence interval)	0.167	0.174	0.0067 (-0.0113, 0.0225)**

*Based on a Mixed Effects model where the fixed effects are treatment, maternal age, maternal education, parity, and cluster-level baseline neonatal mortality ratio; the random effect is study cluster.

**Based on predicted probabilities from the Mixed Effects model; predicted probability of the outcome for each individual was calculated assuming all of them are in group A, then repeated assuming all of them are in group B; difference in the means of these probabilities are presented. 95% confidence interval is based on 1000 bootstrap samples. The bootstrap procedure includes generation of the mixed effects model and calculation of the population standardized difference.
