# Supplementary material for: Community level interventions for pre-eclampsia (CLIP) in India: A cluster randomised controlled trial
Source: Pregnancy Hypertens. 2020 Jul;21:166–75. doi: 10.1016/j.preghy.2020.05.008 (PMC7471838; doi:10.1016/j.preghy.2020.05.008)
Supplement: Supplementary data 6 [file mmc6.docx]

**Supplemental tables**

- Table S1: CLIP Working Group
- Table S2: CLIP triggers and treatment
- Table S3: Characteristics of the intervention
- Table S4: Sensitivity analyses for the primary outcome

**Table S1 CLIP Trials Working Group for appearance on PubMed**

| **First and middle names** | **Last names** |
| --- | --- |
| Shashidhar G | Bannale |
| Keval S | Chougala |
| Vaibhav B | Dhamanekar |
| Anjali M | Joshi |
| Namdev A | Kamble |
| Gudadayya S | Kengapur |
| Uday S | Kudachi |
| Sphoorthi S | Mastiholi |
| Geetanjali I | Mungarwadi |
| Esperança | Sevene |
| Khátia | Munguambe |
| Charfudin | Sacoor |
| Eusébio | Macete |
| Helena | Boene |
| Felizarda | Amose |
| Orvalho | Augusto |
| Cassimo | Bique |
| Ana Ilda | Biz |
| Rogério | Chiaú |
| Silvestre | Cutana |
| Paulo | Filimone |
| Emília | Gonçálves |
| Marta | Macamo |
| Salésio | Macuacua |
| Sónia | Maculuve |
| Ernesto | Mandlate |
| Analisa | Matavele |
| Sibone | Mocumbi |
| Dulce | Mulungo |
| Zefanias | Nhamirre |
| Ariel | Nhancolo |
| Cláudio | Nkumbula |
| Vivalde | Nobela |
| Rosa | Pires |
| Corsino | Tchavana |
| Anifa | Vala |
| Faustino | Vilanculo |
| Rahat N | Qureshi |
| Sana | Sheikh |
| Zahra | Hoodbhoy |
| Imran | Ahmed |
| Amjad | Hussain |
| Javed | Memon |
| Farrukh | Raza |
| Olalekan O | Adetoro |
| John O | Sotunsa |
| Sharla K | Drebit |
| Chirag | Kariya |
| Mansun | Lui |
| Diane | Sawchuck |
| Ugochi V | Ukah |
| Mai-Lei | Woo Kinshella |
| Shafik | Dharamsi |
| Guy A | Dumont |
| Tabassum | Firoz |
| Ana Pilar | Betrán |
| Susheela M | Engelbrecht |
| Veronique | Filippi |
| William A | Grobman |
| Marian | Knight |
| Ana | Langer |
| Simon A | Lewin |
| Gwyneth | Lewis |
| Craig | Mitton |
| Nadine | Schuurman |
| James G | Thornton |
| France | Donnay |
| Romano N | Byaruhanga |
| Brian | Darlow |
| Eileen | Hutton |
| Mario | Merialdi |
| Lehana | Thabane |
| Kelly | Pickerill |

**Table S2: CLIP triggers and treatment**

|  | **Urgent transport** | **MgSO_4_†**  5g IM in each buttock | **Methyldopa‡** 750mg po | **Non-urgent transport** |
| --- | --- | --- | --- | --- |
| **Maternal risk triggers** |  |  |  |  |
| **sBP ≥160mmHg** (consistent with severe pre-eclampsia | ● | ● | ● |  |
| **Unconsciousness*** | ● |  |  |  |
| With sBP ≥160mmHg | ● | ● | ● |  |
| **Recent stroke or seizure** | ● | ● |  |  |
| With sBP ≥160mmHg | ● | ● | ● |  |
| **Significant vaginal bleeding** | ● |  |  |  |
| With sBP ≥140mmHg – presumed abruption and severe pre-eclampsia | ● | ● |  |  |
| **miniPIERS predicted probability ≥25%** | ● | ● |  |  |
| **Fetal risk triggers** |  |  |  |  |
| **No fetal movements in last 12hr** | ● |  |  |  |
| **Heavy proteinuria ≥4+** | ● |  |  |  |
| **None of the 7 triggers and non-severe hypertension** (sBP 140-159mmHg) |  |  |  | ● |

** Unconscious women had to be severely hypertensive before receiving MgSO_4_ and methyldopa, in the event that their unconsciousness was due to another cause unrelated to hypertension, such as obstetric sepsis or hypotension due to bleeding.*

*† MgSO_4_ was administered for eclampsia or severe pre-eclampsia, defined as hypertension that was either severe, or associated with stroke, abruption, or a high probability (≥25%) of an adverse maternal outcome within the next 48hr.*

*‡ Methyldopa was adminsitered only for severe hypertension.*

***Table S3*: CLIP intervention**

| **Components** | **Intervention**  **(n=7839 pregnancies)** |
| --- | --- |
| Community engagement sessions (n) | 1379 |
| Median community engagement sessions by cluster | 214.5 [191.75 – 265.75] |
| Median community engagement participants by cluster | 6742.5 [5736 - 7154] |
| ASHAs and ANMs trained (n) | 148 |
| POM-guided CLIP visits (n) | 63992 |
| POM-guided visits/pregnancy (median [IQR]) | 8.00 [4.00 - 14.00] |
| Antenatal | 7.00 [4.00 - 12.00] |
| Postnatal | 2·0 [1·0, 4·0] |
| Emergency condition identified (and BP not taken) (n (%)) | 5/36 (13·9%) |
| BP measurement (all visits) (n (%)) | 63605 (99·4%) |
| Proteinuria measurement (of first and any hypertensive visit) (n (%)) | 7017/7598 (92·4%) |
| Complete visits that resulted in a recommendation (n (%)) | 63169/63992 (98·7%) |
| Pregnancies with ≥1 POM-guided CLIP visit (n (%)) | 7054/7839 (90·0%) |
| Complete visits (n (%)) | 63706/63992 (99.6%) |
| Pregnancies compliant with POM-guided CLIP visit frequency (n (%)) | 2607 (42·4%) |
| Pregnancies given methyldopa (n (%)) | 61 (0·9%) |
| Accepted (n (%)) | 52 (85·2%) |
| Pregnancies given IM magnesium sulphate (n (%)) | 69 (1·0%) |
| Accepted (n (%)) | 49 (71·0%) |
| Pregnancies referred to facility (n (%)) | 490 (6·9%) |
| Accepted (n (%)) | 427 (87·1%) |

ANMs= Auxiliary Nurse Midwives. ASHAs-=Accredited Social Health Activists. CEmOC=Comprehensive Emergency Obstetric Care. CLIP=Community-Level Interventions for Pre-eclampsia. IM=intramuscular. POM=PIERS-On-the-Move.

***Table S4:* Sensitivity analyses**

| **Sensitivity analysis** | ***Odds ratio (95% CI)*** | ***p-value*** | ***Adjusted*** | ***Imputed*** |
| --- | --- | --- | --- | --- |
| Composite primary outcome | 0·92 (0·74, 1·15) | 0·47 | ✓ | ✓ |
| Unadjusted OR | 0·99 (0·78, 1·24) | 0.94 | ✗ | ✓ |
| Complete postpartum follow up | 0·94 (0·74, 1·19) | 0.60 | ✓ | ✗ |
| Complete responses to all components of primary outcome | 0.94 (0.73, 1.21) | 0.64 | ✓ | ✗ |
| EDD + 3 weeks falls within trial timeline | 0·94 (0·74, 1·19) | 0·58 | ✓ | ✓ |
| EDD + 3 weeks + 42 days falls within trial timeline | 0·94 (0·75, 1·18) | 0·60 | ✓ | ✓ |
| Including only pregnancies with POM visits | 0·96 (0·77, 1·19) | 0·68 | ✓ | ✓ |
| Baseline-adjusted OR | 0·97 (0·71, 1·31) | 0·83 | ✓ | ✓ |
| Maternal mortality | 0·49 (0·094, 2·548) | 0.40 | ✓ | ✗ |
| Maternal morbidity | 0·95 (0·61, 1·50) | 0·84 | ✓ | ✗ |
| Stillbirth and neonatal mortality | 1·09 (0·90, 1·33) | 0·36 | ✓ | ✗ |
| Neonatal morbidity | 0·98 (0·65, 1·48) | 0·93 | ✓ | ✗ |
| Cluster-level aggregate model | 0·99 (0·96, 1·02) | 0·61 | ✗ | ✗ |

*OR=odds ratio. POM=piers on the move. EDD=estimated due date. Adjusted odds ratio presented as odds ratio (95% confidence interval). OR=odds ratio. ✓=yes ✗=no*
